# Sex-dependent alterations of the femoral geometry in a mouse model of Marfan syndrome

**DOI:** 10.1093/jbmrpl/ziag072

**Published:** 2026-04-20

**Authors:** Gina Agostini-Walesch, Tyler Gallagher, Brian Tibble, Anna Stimpson, Mitra Esfandiarei

**Affiliations:** College of Dental Medicine, Midwestern University, Glendale, AZ 85308, United States; Arizona College of Osteopathic Medicine, Midwestern University, Glendale, AZ 85308, United States; Arizona College of Osteopathic Medicine, Midwestern University, Glendale, AZ 85308, United States; College of Graduate Studies, Midwestern University, Glendale, AZ 85308, United States; College of Graduate Studies, Midwestern University, Glendale, AZ 85308, United States; College of Medicine Phoenix, University of Arizona, Phoenix, AZ 85004, United States; Faculty of Medicine, University of British Columbia, Vancouver, British Columbia V6T 1Z3, Canada

**Keywords:** Marfan syndrome, connective tissue disorders, bone rigidity, bone circularity, bone structure

## Abstract

Marfan syndrome (MFS), caused by mutations in the *Fbn1* gene, is associated with skeletal fragility; yet, the geometric and mechanical consequences for long bones remain underexplored. We examined femoral geometry and rigidity in male and female *Fbn1^C1041G/+* (MFS) and WT *C57BL/6 J* (littermate control) mice (*n* = 5/sex/genotype) at 6 mo of age using high-resolution micro-CT. Femur length, cortical and medullary areas, cross-sectional circularity, and derived rigidity indices (*I*_MAX_, *I*_MIN_) were quantified across 3 diaphyseal locations. Marfan syndrome mice had significantly longer femora than controls, with more circular cross-sections regardless of sex. Male MFS mice displayed endosteal expansion, reduced cortical bone (−18% to 30%), and lower bending rigidity, indicating weakened structural resistance. In contrast, female MFS mice maintained or exceeded control rigidity values despite geometric alterations. These findings reveal pronounced sex-dependent skeletal effects in MFS, suggesting that cortical bone loss and estimated mechanical weakening are male-biased features, potentially linked to hormonal influences and altered bone remodeling. Such differences may contribute to differential fracture risk and have implications for targeted interventions in connective tissue disorders.

## Introduction

Marfan syndrome (MFS) is a heritable connective tissue disorder caused by mutations in the *FBN1* gene, which encodes fibrillin-1 (Fbn1), a critical extracellular matrix glycoprotein involved in the formation of elastic fibers and the regulation of multiple signaling cascades, particularly the transforming growth factor-β (TGF-β) signaling. While MFS is classically defined by cardiovascular, ocular, and skeletal manifestations, the extent and impact of skeletal fragility remain incompletely understood. Notably, individuals with MFS often exhibit signs of bone weakness, such as reduced BMD and increased fracture susceptibility, particularly in weight-bearing bones.^1–3^

Studies suggest that Fbn1 plays a key structural role in bone matrix organization and influences bone integrity by modulating osteoblast differentiation and matrix mineralization through the regulation of TGF-β activity.^4^^,^^5^ Critically, TGF-β suppresses osteoblast differentiation, in part, by inhibiting the master osteogenic factor Runx2.^4^ Mechanistically, TGF-β/SMAD2/3 signaling reduces Runx2 stability (eg, via ubiquitin-mediated turnover), thereby limiting osteoblast maturation, mineral deposition, and bone formation, emphasizing the role of TGF-β in regulating osteoblast function. In MFS, abnormal Fbn1 augments TGF-β signaling, promoting Runx2 inhibition and shifting the balance away from osteoblast differentiation toward dysregulated mineralization, ectopic bone formation, and impaired skeletal integrity.^6^

In both patients with MFS and experimental mouse models, distinct alterations in limb bone architecture have been well documented. These include tall stature and excessive limb bone growth, which are thought to result from the loss of locally mediated TGF-β inhibition caused by Fbn-1 deficiency.^7^ Additionally, deficits in bone mineralization are consistently observed, beginning in childhood and persisting into late adulthood,^1^^,^^8–11^ though some caution is warranted given the wide variation in measurement techniques and sampling across studies.^12^

The specific geometric and mechanical consequences of MFS, especially for long bones like the femur, are less well-characterized.^6^^,^^13^ Parameters such as cross-sectional shape and the distribution of cortical bone (within it) can offset mineral declines to maintain bone rigidity, however, such properties remain underexplored in the context of sex-specific phenotypes and disease progression. Furthermore, persistent methodological issues limit the interpretability of prior studies, including inconsistent size controls, the pooling of sexes, the omission of age restrictions or covariates, and a reliance on absolute rather than relative sampling sites for cortical and trabecular bone measurements.^8^^,^^11^^,^^14^ Because of allometric effects, sex- and MFS-related variation in bone length, and the established influence of sex and age on bone development and remodeling, such limitations make it difficult to detect more nuanced (yet meaningful) structural changes within MFS phenotypes and their likely consequences, including those that may be sex-specific. The present study is designed as a phenotypic characterization of femoral diaphyseal shape and geometry-derived indices of structural rigidity in Fbn1^C1041G/+ mice. Our primary aim is to quantify sex-dependent differences in bone structural morphology at a single adult time point. This work is not intended to resolve the molecular or cellular mechanisms underlying the observed phenotypes.

To address the existing gaps in knowledge, we completed a detailed analysis of the structural properties of the femur—the longest, heaviest, and strongest bone in the mouse skeleton—in both male and female MFS (*Fbn1^C1041G/+*) and control C57BL/J mice at 6 mo of age, a time point when characteristic phenotypic changes, including kyphosis and aortic root aneurysm, are typically evident. Animal models harboring *Fbn1* mutations, such as *Fbn1^C1041G/+* and *mg^Δlpn^*, have proven valuable in dissecting MFS-related skeletal abnormalities, including reduced trabecular thickness and altered cortical composition.^3^^,^^15^ In the *Fbn1^C1041G/+* mouse model used in this study; the missense variant replaces a conserved cysteine within a calcium-binding EGF-like (cbEGF) domain, disrupting the disulfide-bonding pattern required for proper folding, secretion, and assembly of fibrillin-1 into extracellular microfibrils. The result is poor incorporation of mutant fibrillin, leading to production of fewer and abnormal microfibrils, compromising the sequestration of latent TGF-β and thereby increasing TGF- β bioavailability.^16^

In this study, our analysis focused on the precise quantification of maximum femoral length, the distribution of cortical bone in cross-section, diaphyseal shape, and derived rigidity metrics (ie, estimated mechanical consequences) based on micro-CT (mCT) data. The main objective of this study is to define how these parameters vary with genotype and sex in an age-controlled sample. By identifying subtle architectural deviations that may compromise bone integrity, this study seeks to enhance understanding of the skeletal phenotype in MFS and related connective tissue disorders and establish early indicators of mechanical vulnerability.

## Materials and methods

### Experimental animal model and bone sample preparation

Male and female MFS (*Fbn1^C1041G/+*) mice and their WT littermate controls (*Fbn1^+/+*) were used for this study (*n* = 5/group). WT controls were littermates generated from the same breeding scheme and were on a C57BL/6 background. All animals were examined at 6 mo of age, a time point chosen to reflect kyphosis and full aortic root aneurysm progression and early adulthood, with the aim of capturing phenotypic changes prior to age-related skeletal degeneration. All animal care and experimental procedures were conducted according to the National Research Council Guide for the Care and Use of Laboratory Animals and the Guidelines for Animal Experiments and the Midwestern University Animal Care and Use Committee [IACUC protocols AZ-4182]. Mice were group-housed (up to 5 mice per cage) in a 12/12 h light-dark cycle with food and water available ad libitum. At 6 mo of age, mice were euthanized using 5% isoflurane inhalation followed by cervical dislocation.

Specimens were stored frozen at −20° freezer and then express shipped on dry ice to the University of Arkansas MicroCT Imaging Consortium for Research and Outreach for mCT scanning. Sample transport and return followed all legal requirements, best standard practices for shipping biological samples, and the Jackson Laboratory Material Transfer Agreement. At the time of scanning, whole-body specimens were prepared for mCT by placing them in individual 50 mL conical vials, with the limbs separated using low-density foam to facilitate accurate digital segmentation.

### Micro-CT parameters

The caudal half of each specimen was scanned in its container using a Nikon X TH 225 ST mCT scanner (Nikon, Tokyo, Japan) to produce Tag Image File Format stacks with a mean isotropic voxel size of 0.02 ± 0.005 depending on specimen size, a resolution is sufficient to capture even minute elements of bony architecture, such as individual trabecular struts.^17^

### Shape and mechanical data extraction

**Figure 1:**
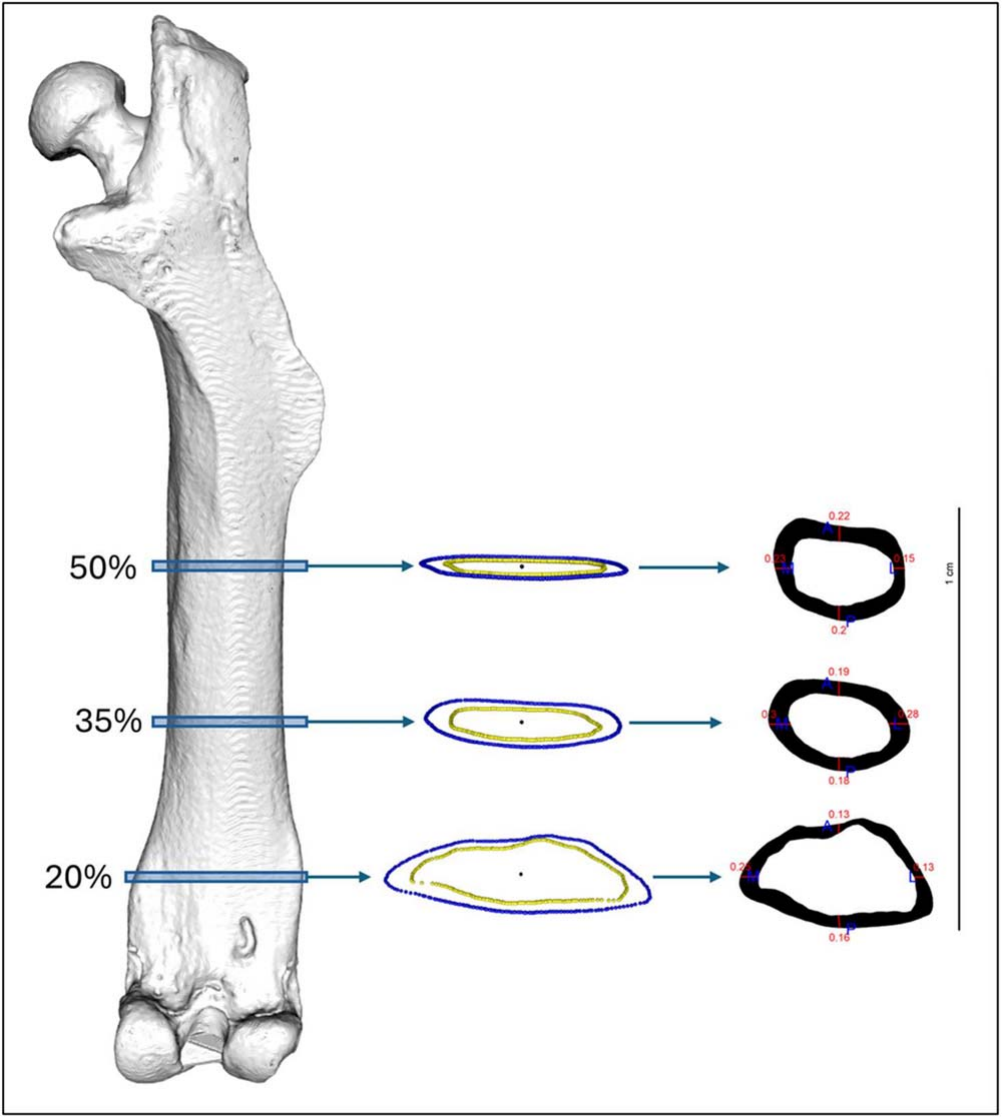
Mesh-derived cross-sectional slices used in this study, including distal (20%), mid-distal (35%), and midshaft (50%). Red numbers reflect cortical thickness (mm). A = anterior (rostral); *p* = posterior (caudal); M = medial; and L = lateral (female, MFS phenotype).

To test differences in femoral shape and structure between phenotypes, 3D Slicer^18^ was used to segment and extract the femoral periosteal and endosteal 3D meshes from mCT data (Figure S1). Meshes for all samples were then aligned to a standard orientation following Profico et al.,^19^ and the maximum length was calculated (Table 1). Afterwards, the distal, mid-distal, and midshaft diaphyseal cross-sectional slices were extracted at 20%, 35%, and 50% maximum bone length using the R package morphomap (Figure 1) as previously described^19^ using R Software Program (https://www.r-project.org/). Proximal regions were avoided due to the presence of branching structures, including the lesser and greater trochanters, the FN, and gluteal insertions (third trochanter). Additionally, mechanical manipulation studies have shown that distal regions in mice are more mechanosensitive,^20^ with fractures most commonly occurring at the midshaft.^21^

**Table 1:** Variables measured in the present study.

**Variable**	**Abbr.**	**Raw units**	**Criteria**
**Maximum length**	N/A	mm	Maximum length of the femur measured as the linear distance between proximal-greater trochanter and distal-most medial condyle
**Total area**	Tt.Ar	mm^2^	Total area enclosed by the periosteal boundary
**Medullary or marrow area**	Ma.Ar	mm^2^	Total area enclosed by the endosteal boundary
**Cortical area**	Ct.Ar	mm^2^	Total amount of cortical bone present in the cross-sectional slice
**Percent cortical bone**	%CA	NA	The relative amount of cortical bone present in the cross-sectional slice as a proportion of total area
**Maximum second moment of area**	*I*_MAX_	mm^4^	The maximum bending rigidity based on slice geometry
**Minimum second moment of area**	*I*_MIN_	mm^4^	The minimum bending rigidity based on slice geometry
**Circularity**	AP:ML	NA	A ratio between the AP and ML axes measured as the diameter between periosteal margins

Abbreviations: AP, anteroposterior; ML, mediolateral.

Based on those previous studies, we used mouse femora, as they are the largest bones in the mouse skeleton and exhibit pronounced mechanosensitivity, in some cases exceeding that of the tibia.^22^ We did not include trabecular bone in our analysis, given its relatively limited and inconsistent relationship with bending rigidity in diaphyseal regions.^23–26^ Analyses were focused on the *diaphyseal cortex*, because diaphyseal bending and torsional resistance are primarily determined by the distribution of cortical bone about the longitudinal axis. Trabecular outcomes were not quantified because the study did not target epiphyseal/metaphyseal regions, where trabecular architecture predominates, and because the primary study outcomes are derived from diaphyseal cross-sectional geometry. We interpret “slenderness/gracility” within the diaphysis as being reflected by total cross-sectional area and external shape metrics, which are reported here alongside rigidity indices.

Cross-sections were sampled at standardized locations expressed as a percentage of femur length to ensure anatomical comparability across individuals. Because second moments of area are defined for 2D cross-sectional geometry, values were computed from individual cross-sections at each location. For each cross-sectional slice, geometric parameters (eg, minimum and maximum diameters, the cortical area, and the medullary area) along with derived strength indices were calculated. Second moments of inertia (I) are section-specific parameters that utilize cross-sectional geometry to quantify a bone’s capacity to resist deformation under bending, a common loading condition experienced by long bones in vivo*.*^21^ Two common “I” metrics, I_MAX_ and I_MIN_ represent the maximum and minimum resistance of a bone to unidirectional bending in a given slice and serve as general indicators of mechanical strength based on the distribution of cortical bone about the neutral axis.^27^ This measure is independent of material properties and, therefore, should be viewed as an estimate of bone rigidity. However, abundant ex vivo research shows that the distribution of cortical bone in cross-section is one of the strongest indicators of a diaphyseal bone’s compressive, axial, and bending rigidity and strength, often to a greater effect than density.^28^^,^^29^ While we used a single slice to extract our “I” values, another recent study used the mean value of multiple slices reflecting a 5%-10% region of bone by length.^3^ We therefore conducted a supplemental test confirming there was no significant difference in I_MAX_ calculated via a single slice (our approach) and I_MAX_ calculated as the mean of 7 slices taken at 1% intervals centered on the midshaft for our samples (reflecting a 6% region of bone by length). These data are presented in Table S1.

Limb bone structure is influenced by size through both allometric effects and variations in baseline mechanical loading related to BW.^21^ Since live body mass and length data were not available for all specimens, centroid size was derived from 17 periosteal mesh landmarks consistently applied uniformly to all samples using the ALPACA extension for 3D Slicer.^30^ Linear models (LM) were then used to assess the fixed effects of sex, phenotype, their interaction, size, and cross-sectional slice location on the variables listed in Table 1.

To visualize differences in cross-sectional shape, 2D coordinate data representing the midshaft periosteal perimeter in each slice were extracted and subjected to Procrustes transformation.^31^ The consensus (mean) shape for each sex and phenotype^32^ was then calculated using the R *package geomorph* (https://cran.r-project.org/web/packages/geomorph/index.html), and plotted to facilitate visual comparisons between groups.

### Statistical analyses

All statistical analyses were performed using R software, version 4.4.3 (R Core Team, Vienna, Austria). Data were first inspected for normality using the Shapiro-Wilk test (>.05) and by visual assessment of Quintile-Quintile plots. Extreme outliers for *I*_MAX_ (2 outliers), *I*_MIN_ (3 outliers), and percent cortical area (3 outliers) were identified as values lying beyond 3 times the interquartile range and removed (<3% of the total dataset). Analyses were hypothesis-driven and focused on testing the effects of genotype, sex, and their interaction (with size adjustment) on cross-sectional geometry and rigidity indices. The goal was not to develop a multivariable predictive model; therefore, stepwise model selection was not used.

To assess the effects of Genotype (*Fbn1^C1041G/+* vs *Fbn1^+/+*), sex (male vs female), and their interaction on morphometric and mechanical variables, LM, and 2-way ANOVA were used, with centroid size included as a covariate to account for variation in body size when appropriate. Slice location (20%, 35%, and 50% of bone length) was included as a fixed factor in models of cross-sectional and strength parameters. Model explanatory power was evaluated using the adjusted coefficient of determination (adjusted *R*^2^).

For shape analyses, periosteal perimeter coordinates from the midshaft were subjected to generalized Procrustes alignment, and consensus shapes were calculated for each sex-phenotype combination using the geomorph R package (v. 4.0.10). Differences in cross-sectional geometry were evaluated using ANOVA on Procrustes-aligned coordinate data. Statistical significance was set at *α* = 0.05, and all *p* values are reported as 2-tailed. When multiple pairwise comparisons were performed, *p*-values were adjusted using the Holm-Bonferroni method to control for type I error. Graphical outputs were generated using the *ggplot2* and *geomorph* packages in R.

## Results

### Male and female MFS mice have significantly longer femora than controls

A 2-way ANOVA revealed that both male and female MFS mice had significantly longer femora than controls (Figure S2; *p* < .001, *F* = 59.243) with no significant interaction between sex and phenotype (*p* = .0542, *F* = 4.277) or main effect of sex (*p* = .403, *F* = 0.735). Control males (16.11 ± 0.25 mm) and females (16.07 ± 0.18 mm) had shorter femora than MFS males (16.69 ± 0.28 mm) and females (16.97 ± 0.22 mm).

### MFS male mice have proportionately less cortical bone, due to enlarged medullary areas

**Figure 2:**
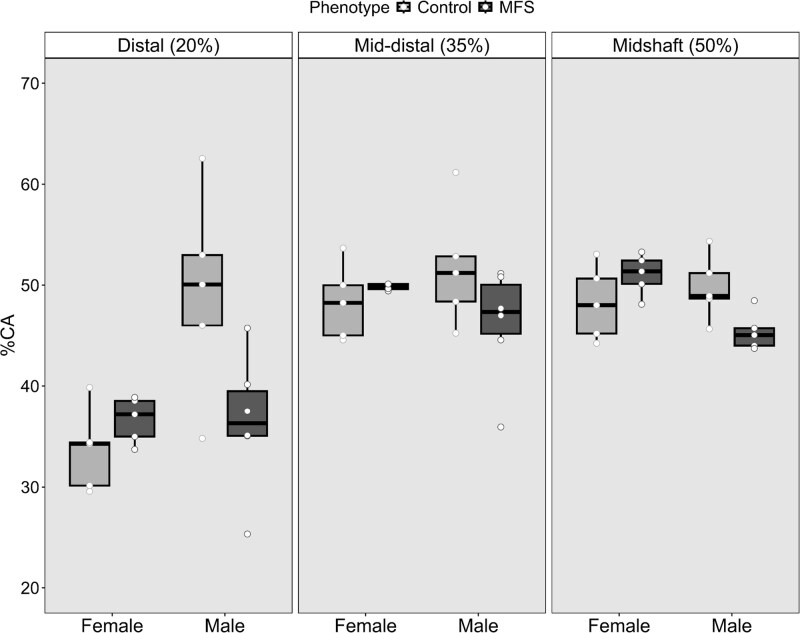
Differences in the percentage of each slice containing cortical bone. MFS = Marfan syndrome phenotype; horizontal black bar = median. A significant interaction between sex and phenotype was reported for cortical bone area after controlling for size and slice location (*p*-value < .001, see Table 2).

Data presented in Table 2 summarize the results for the area measures examined: subperiosteal (total) area, medullary area, and cortical bone area. All 3 models demonstrated moderately high to high explanatory power, accounting for 63.2%-89.8% of the variance. For total area males had larger cross-sectional areas than females, but no phenotype differences were detected within sex. In contrast, both medullary and cortical bone area showed significant sex-by-phenotype interactions. Marfan syndrome males exhibited relatively larger medullary areas surrounded by less cortical bone—a pattern not observed in females. After adjusting for size, this corresponded to an 18%-30% reduction in cortical bone amount for MFS males (Figure 2).

**Table 2:** Regression results and area measurements in mice.

**Fixed effects**	**Total area**			**Medullary area**			**Cortical area**		
	**Estimate**	**95% CI**	***p*-value**	**Estimate**	**95% CI**	***p*-value**	**Estimate**	**95% CI**	***p*-value**
**Intercept**	1.190	−2.08 to 4.45	.477	−0.480	−3.84 to 2.89	.780	1.340	−1.06 to 3.75	.274
**Sex**	0.390	0.25–0.54	**<.001**	0.010	−0.14 to 0.16	.890	0.280	0.15–0.41	**<.001**
**Phenotype**	0.080	−0.05 to 0.20	.216	−0.010	−0.14 to 0.12	.871	0.070	−0.02 to 0.16	.150
**Slice, 35%**	−0.990	−1.09 to −0.88	**<.001**	−0.830	−0.94 to −0.71	**<.001**	0.110	−0.10 to 0.31	.321
**Slice, 50%**	−1.010	−1.11 to −0.90	**<.001**	−0.810	−0.92 to −0.70	**<.001**	0.080	−0.13 to 0.29	.475
**Size**	0.050	−0.05 to 0.15	.324	0.070	−0.03 to 0.17	.183	−0.030	−0.10 to 0.04	.379
**Total area**	—	—	—	—	—	—	0.270	0.07–0.47	**.007**
Sex*Phenotype	−0.170	−0.35 to 0.02	.073	0.200	0.01–0.39	**.037**	−0.320	−0.46 to −0.19	**<.001**
**Model strength**									
***R*** ^**2**^ **(adjusted)**		**0.898**			**0.849**			**0.632**	

Total area added to model for cortical area following Jepsen et al.^21^; ref = female, control, 20% slice.

### MFS mice have more circular cross-sectional geometries in a sex-independent manner

**Figure 3:**
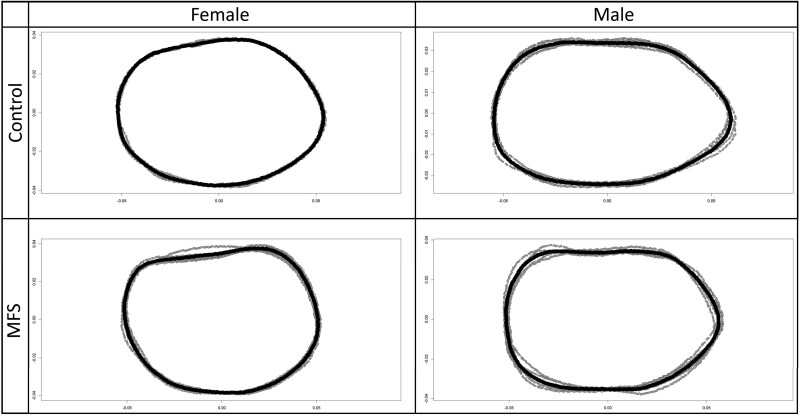
Periosteal midshaft consensus shape differences by sex and phenotype. Some ML loss is present in both sexes, thereby bringing the AP:ML ratio closer to 1.0 (a more circular perimeter). However, ML losses are significantly greater in males. Periosteal contours have been size-scaled. Gray lines = individual periosteal contours; black line = consensus (mean) contour. Top margin = anterior/rostral, left margin = lateral.

**Figure 4:**
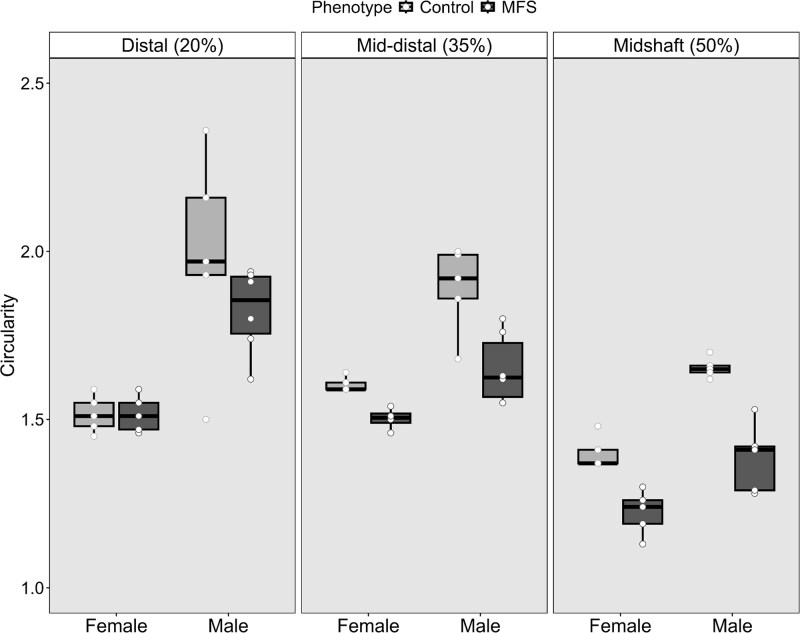
Differences in cross-sectional circularity as reflected by AP:ML ratio. MFS = Marfan syndrome phenotype; horizontal black bar = median. AP:ML ratio significantly differed by sex (*p*-value < .0001) and by phenotype (*p*-value = .044, see Table 3) after controlling for size and slice location.

We measured the differences in cross-sectional circularity (a ratio between the anteroposterior [AP] and mediolateral [ML] principal diameters) in male and female control and MFS mice (Figure 3). Both male and female MFS mice exhibited more circular cross-sections at all measured locations (Table 3; Figure 4; *p* < .0001, *F* = 46.08), with the effect being more pronounced in males. The model demonstrated high explanatory power, accounting for 72.6% of the variance. It is important to note that the data presented in Tables 2 and 3 summarize group comparisons of geometry and rigidity outcomes (with size adjustment), rather than identifying a “best predictor” model across multiple competing variables.

**Table 3:** Regression data, minimum, and maximum bone rigidity in mice.

**Fixed effects**	***I***_**MIN**_			***I***_**MAX**_			**AP:ML ratio**		
	**Estimate**	**95% CI**	***p*-value**	**Estimate**	**95% CI**	***p*-value**	**Estimate**	**95% CI**	***p*-value**
**Intercept**	0.300	−0.17 to 0.76	.209	−0.140	−2.25 to 1.97	.897	1.625	1.560–1.691	**<.0001**
**Sex (ref = F)**	0.050	0.03–0.07	**<.001**	0.310	0.21–0.40	**<.001**	0.336	0.260–0.412	**<.0001**
**Phenotype (ref = control)**	0.030	0.01–0.04	**.004**	0.050	−0.03 to 0.13	.215	−0.094	−0.170 to 0.018	**.044**
**Slice, 35%**	−0.100	−0.11 to −0.08	**<.001**	−0.340	−0.41 to −0.27	**<.001**	−0.055	−0.119 to −0.010	*NS*
**Slice, 50%**	−0.080	−0.09 to −0.06	**<.001**	−0.430	−0.50 to −0.36	**<.001**	−0.301	−0.366 to −0.237	**<.0001**
**Size**	0.000	−0.02 to 0.01	.725	0.020	−0.04 to 0.08	.480	−0.129	−0.235 to −0.024	**.045**
Sex*Phenotype	−0.050	−0.08 to −0.03	**<.001**	−0.190	−0.31 to −0.07	**.002**	−0.170	−0.35 to 0.02	.073
**Model strength**									
***R*** ^**2**^ **(adjusted)**		**0.784**			**0.801**			**0.726**	

Abbreviations: AP, anteroposterior; ML, mediolateral; ref = female, control, 20% slice.

### MFS male mice have lower resistance to bending force while female MFS mice are similar to or higher than controls

**Figure 5:**
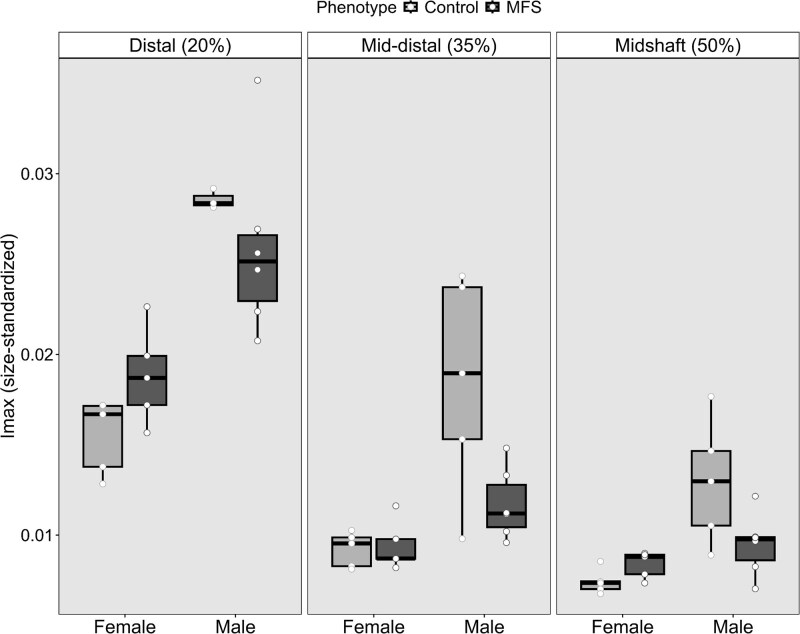
Differences in size-standardized *I*_MAX_ by sex and phenotype. MFS = Marfan syndrome phenotype; horizontal black bar = median. A significant interaction between sex and phenotype was reported for *I*_MAX_ after controlling for size and slice location (*p*-value = .002, see Table 3).

Regression analysis revealed significant effects of the sex-by-phenotype interaction and slice location on both maximum and minimum rigidity (Table 3), with high explanatory power (80.1% and 78.4% of the variance explained, respectively). Overall, males exhibited higher maximum rigidity than females (Figure 5); however, MFS males had significantly lower maximum rigidity than male controls across all 3 bone sections evaluated. In contrast, MFS females maintained or even exceeded the maximum rigidity of female controls (Figure 5). As expected, maximum rigidity was greatest near the knee, where cross-sectional areas were largest (Figure S3). For minimum rigidity, MFS females showed slightly higher values, suggesting greater resistance to bending stresses, whereas the opposite trend was observed in males (Figure S3). Collectively, these findings indicate that MFS males have weaker bones overall—both in terms of their highest and lowest unidirectional bending force tolerances.

## Discussion

This study provides a comparative assessment of femoral diaphyseal geometry and geometry-derived rigidity indices in a Marfan mouse model, with an explicit evaluation of sex as a biological variable. Because our analyses are based on micro-CT-derived morphology, cellular mechanistic inferences are intentionally limited. We note that recent work has examined skeletal phenotypes in the same Marfan model. The present study complements this literature by focusing on *size-standardized cross-sectional geometry* at multiple diaphyseal locations and by enabling direct tests of *sex × genotype interactions*.

The findings of our study indicate that MFS in mice is associated with structural alterations of the femur, with effects being more pronounced in males than in females. Significant interactions between sex and phenotype were observed for nearly all variables measured. While maximum and minimum bending rigidities in MFS females were generally comparable to or greater than those of control females, MFS males exhibited femurs that were significantly weaker than those of control males. If these deficits in structural strength are not compensated by changes in material properties, such as bone density,^33^ they are likely to result in a disproportionately elevated fracture risk in MFS males relative to non-MFS males. Individuals with MFS exhibit elevated fracture rates from childhood into adulthood.^34^ In a high-quality retrospective cohort spanning the lifespan, the highest incidence rate ratio was observed in males <20 yr with MFS, followed by females ≥55 yr; however, the association in older women lost significance after excluding those with confirmed osteoporosis.^11^

Our results also show that, although total subperiosteal area does not differ between genotypes, the organization of cortical bone within that area is altered. Marfan syndrome male mice display endosteal expansion accompanied by reduced cortical bone and uniformly thinner cortices. This pattern may reflect an imbalance in the normal coupling of bone deposition and resorption on the endosteal surface, favoring removal at a rate exceeding replacement. Such a remodeling profile resembles that observed with advanced age^35^ and, more specifically, in women undergoing menopause, where estrogen decline accelerates endosteal resorption without sufficient compensatory subperiosteal apposition.^36^

Over time, this imbalance lowers the amount and density of cortical and trabecular bones in cross-section, producing structural weakening and an increased risk of bone fracture. Similar effects are seen in men, whose early-adulthood bone accrual and late-adulthood bone loss are tightly correlated to circulating estrogen levels with low contributions of testosterone.^37^ Large retrospective studies show that aging men with low bioavailable estrogen concentrations had significantly weaker arm and leg bones characterized by reduced cortical area, cortical thinning, and endosteal expansion, much like postmenopausal women.^38^ Furthermore, men with estrogen receptor or aromatase deficiencies have skeletal phenotypes characterized by delayed epiphyseal union, above-average height, overactive bone remodeling, and low density (particularly of cortical bone), characteristics consistent with those observed in our data. Evidence on estrogen deficiency in premenopausal women is limited and methodologically weaker, with mixed results.^39^

In our MFS model, increased cross-sectional circularity coupled with cortical thinning indicates overall bone weakening, reflecting a loss of material along the ML axis with insufficient compensatory gains AP. Such weakening may arise from multiple factors, including intrinsic influences such as changes in bending patterns, hormonal differences that alter mechanosensitivity, reduced habitual mechanical loading, or local strain due to muscle weakening or disuse.^40^ Beyond its structural effects on bone, estrogen deficiency has also been associated with diminished muscle function, recovery capacity, and mass in both men and women.^41^^,^^42^ This muscle-related decline would further reduce the in vivo strains experienced by bone already predisposed to loss through estrogen-mediated resorption, compounding the structural deficits described above.

Unloading studies show that even modest (20% of BW), short-term levels of unloading are associated with significant reductions in muscle mass, trabecular and cortical bone amount, and bone density, with a disproportionate effect on cortical bone.^43^^,^^44^ Human studies show individuals with MFS have comparably weaker muscles and lower bone density after controlling for body mass, and that the gap between non-MFS and MFS is evident by early childhood and increases into adulthood.^45^^,^^46^ Additionally, human data reveal several anatomical and clinical features in individuals with MFS that could reduce proximal leg loading. These include movement-limiting symptoms such as pain, numbness, joint stiffness, and a restricted range of motion associated with myopathy, as well as structural abnormalities like dural ectasia, poor articulation between the femoral head and acetabulum (protrusio acetabuli), generalized joint laxity, and early onset osteoporosis, which together could substantially diminish the mechanical stimuli necessary for maintaining bone strength in the proximal leg.^47^^,^^48^ These factors, combined with reduced pulmonary capacity and an elevated risk of aortic rupture, lead many clinicians to advise individuals with MFS to avoid moderate-to-high-intensity physical activities, the very types of loading that most effectively challenge the musculoskeletal system and promote bone deposition.^49^

In a study of only female mice by *Zimmermann* et al.*,*^3^ a 17%-19.5% lower tibial *I*_MAX_ was detected, which is opposite to the trend observed for female femora in our dataset. This discrepancy may reflect differences in the target bone examined or the study design, as *Zimmermann* et al. primarily compared structural differences across age cohorts of 10, 26, and 52 wk. In contrast, mice in our study were sacrificed at 34.4 ± 3.9 wk, corresponding to middle adulthood.^50^ However, the more probable explanation is that our analyses used size-standardized data to minimize size bias, whereas *Zimmermann* et al.^3^ reported unstandardized measurements. It is therefore unclear to what extent the structural weakness reported for MFS females in *Zimmermann* et al.^3^ reflects true pathological differences versus effects driven by body size and the associated changes in mechanical loading environments.

This consideration is particularly important given the well-documented body size differences between MFS and non-MFS individuals: people with MFS are, on average, taller than those without the condition, with this trend established early in development.^1^ Increased stature alone can influence derived mechanical values by raising the aspect ratio, even when body mass is comparable to controls.^51^ While such size effects are of general biomechanical interest, they are predicted under standard models and do not necessarily provide mechanistic insight. To draw meaningful conclusions about the specific effects of MFS on limb bone structure, particularly at molecular or cellular levels, size variation should be accounted for, either by standardizing the data or explicitly including size as a covariate in the analytical model.^21^

Although molecular mechanistic measures were not collected in this study, several testable hypotheses may explain the observed sex-dependent structural differences. Sex hormones can modulate bone modeling and remodeling, potentially influencing periosteal apposition and endosteal expansion. In addition, sex differences in growth trajectories and habitual loading may interact with altered extracellular matrix integrity in Fbn1 mutants to produce divergent geometric adaptation. Regional differences along the femur are also plausible; however, because our measurements were limited to defined diaphyseal locations and did not include molecular mapping, regional variation in Fbn1-related signaling cannot be evaluated here and remains an important area for future work.

This is not the first study to identify sex-specific differences in the physical manifestations of MFS, with disproportionately greater effects observed in biological males. For example, although males generally have an elevated risk of thoracic and abdominal aortic aneurysms, this risk is even higher in MFS males due to anatomical characteristics of the aortic root, which, while enlarged in all individuals with MFS, is disproportionately enlarged in males.^52^^,^^53^ Furthermore, although male and female mice with the MFS genotype exhibit similar early patterns of aortic development, their trajectories diverge at later stages: females show a lag in medial collagen accumulation and dilation, a trend described as “*protective.*”^54^ In contrast, MFS males often show more severe aortic phenotypes, including greater elastic fiber damage.^52^^,^^55^ Notably, recent work demonstrated that subcutaneous delivery of 17β-estradiol significantly reduced aortic rupture rates and slowed aortic root growth in young MFS mice, with the most pronounced benefits occurring in males.^53^ In another study, using the Fbn1^mgR/mgR^ MFS mouse model, *Altinbas* et al.^14^ reported reduced TGF-β signaling in males compared with age-matched females, reported reduced TGF-β signaling in males compared with age-matched females.^7^

## Conclusion

As a conclusion, our findings indicate sex-dependent differences in Marfan-associated alterations in femoral diaphyseal geometry and geometry-derived rigidity indices. These results motivate future work integrating whole-bone mechanical testing and molecular assays to determine the extent to which these morphology-based differences translate to functional outcomes and to identify underlying mechanisms.

Several reported Marfan-associated skeletal features in this study are directionally consistent with prior studies; however, important differences in study design affect interpretation. In the present analyses, cross-sectional outcomes and rigidity indices were evaluated with an explicit adjustment for overall size, which is critical when body size and bone length differ between groups. In addition, the inclusion of both males and females enabled direct testing of sex-dependent genotype effects, an analysis not possible in single-sex designs. These design features allow us to separate differences in cross-sectional geometry from differences that may arise indirectly through size- or growth-related variation.

Overall, these findings establish a sex-stratified morphological baseline for femoral diaphyseal geometry in this Marfan mouse model and motivate follow-up studies combining micro-CT with whole-bone mechanical testing, histomorphometry, and molecular assays to link structure to function and mechanism, and to determine whether sex-dependent geometric differences translate to functional differences in whole-bone performance.

## Study limitations

This study examined geometric changes rather than material properties (eg, density), which can have substantial effects on strength if they differ consistently between phenotypes. Therefore, follow-up studies using 3- or 4-point-bend testing or other material tests of bone strength are recommended. Cross-sectional geometry and bone material are interrelated, as geometry can structurally compensate for material deficiencies by positioning cortical bone farther from the neutral axis^56^; however, such compensatory geometry was not observed in our data. Another limitation is that, while mouse cortical bone remodels in response to mechanical stress, mice lack the more complex Haversian-based remodeling seen in larger, longer-lived species such as humans.^10^ This difference does not preclude the use of mice as a model for studying the interplay between genotype, mechanical stress, and bone structure, but it likely entails differences in the rate and magnitude of mechanically induced structural changes between species. Accordingly, future investigations of limb bone structural variation in humans with MFS would provide a valuable complement to the present work. Future studies could extend the present work by integrating trabecular microarchitecture at joint-adjacent regions; however, the present study was designed specifically to address diaphyseal geometry and geometry-derived structural indices.

In addition, we report geometry-derived indices of structural rigidity rather than results from whole-bone mechanical testing. Although these indices provide a morphology-based estimate of structural resistance, they do not directly measure failure properties (eg, strength, toughness) or tissue-level material behavior.

Another limitation of the present study is that we did not collect molecular, histologic, or cell-based measures that would allow direct testing of mechanistic pathways implicated in Marfan bone biology (eg, transforming growth factor beta, matrix metalloproteinases, and ROS). Accordingly, references to candidate pathways are presented as hypotheses informed by prior literature rather than conclusions supported by the present dataset.

## Supplementary Material

Agostini_et_al_Supplementary_Figures_and_Tables_ziag072

## Data Availability

The datasets generated and/or analyzed during the current study are available from the corresponding author on reasonable request.
