# Supplementary material for: Sex-dependent alterations of the femoral geometry in a mouse model of Marfan syndrome
Source: JBMR Plus. 2026 Apr 20;10(6):ziag072. doi: 10.1093/jbmrpl/ziag072 (PMC13171039; doi:10.1093/jbmrpl/ziag072)
Supplement: Agostini_et_al_Supplementary_Figures_and_Tables_ziag072 [file agostini_et_al_supplementary_figures_and_tables_ziag072.pdf]

Supplementary Figure S1

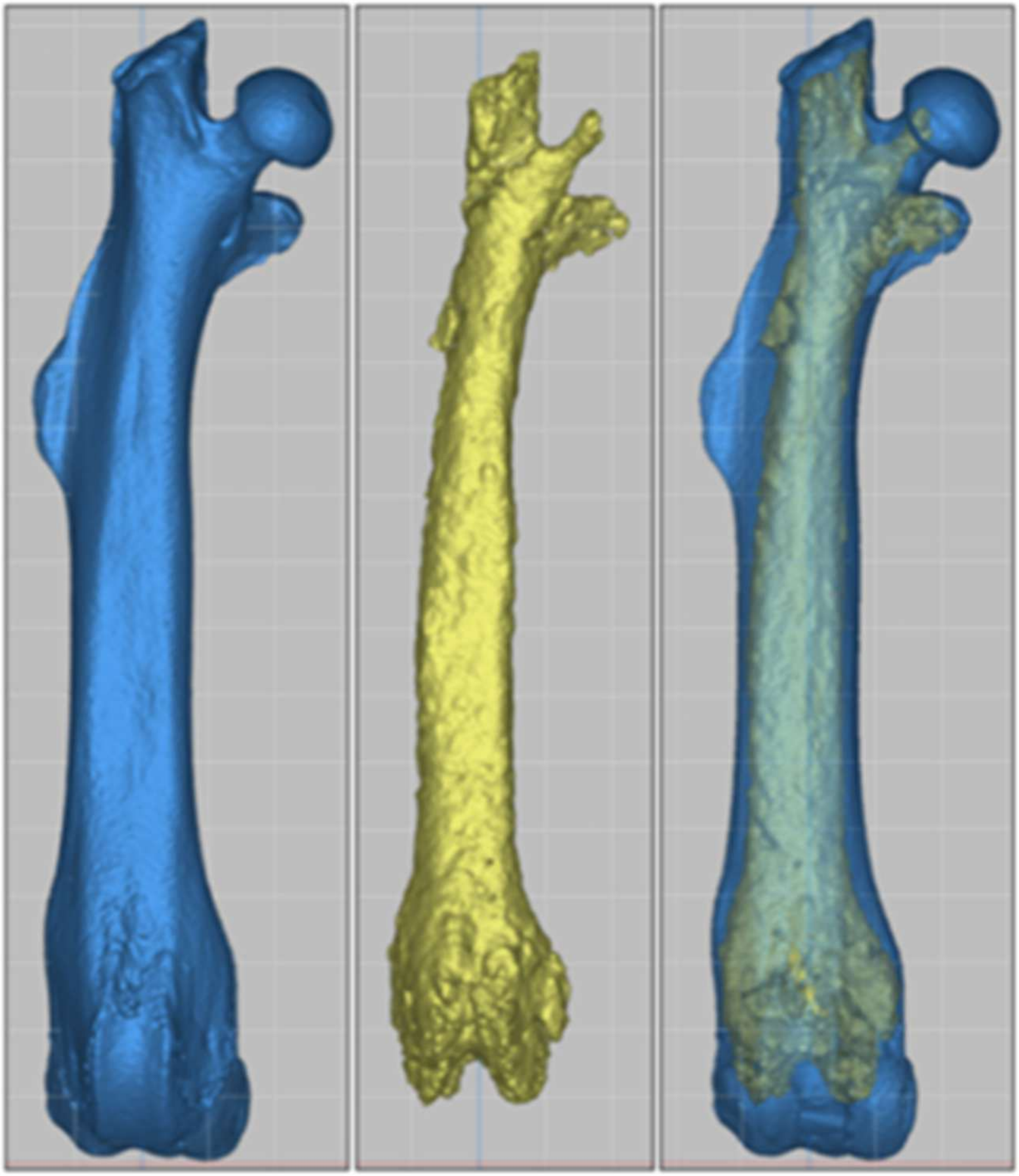

Figure S1. Representative 3D reconstructions of periosteal (blue) and endosteal (yellow) surfaces, along with the merged composite mesh, from a female mouse with the Marfan Syndrome (MFS) phenotype. These reconstructions illustrate the external and internal cortical boundaries used in morphometric and biomechanical analyses.

Supplementary Figure S2

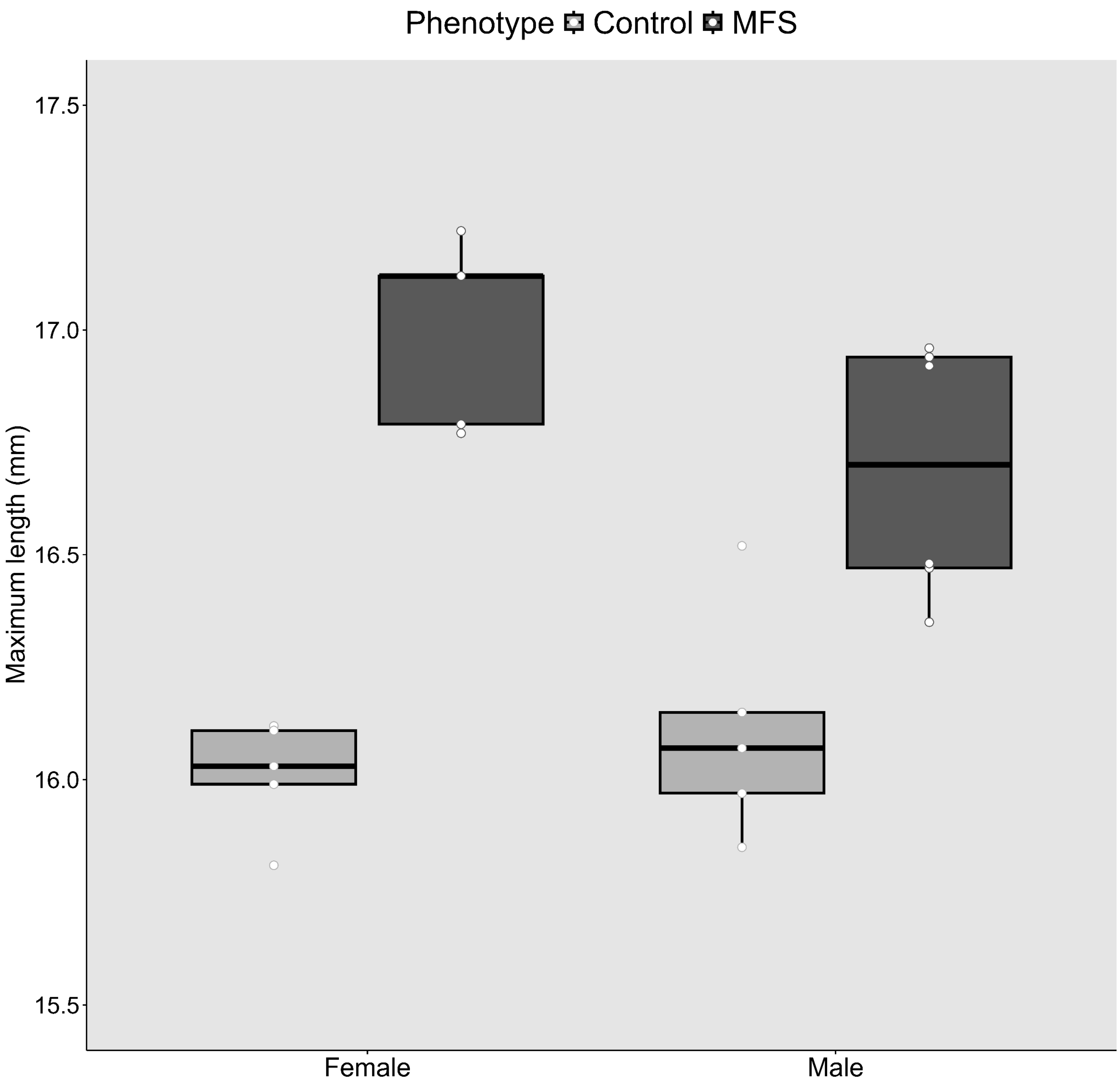

Figure S2. Maximum femoral length by sex and phenotype. Both male and female MFS mice have significantly longer femora than controls ($p < 0.001$, $F = 59.243$). Black bars = medians. No significant interaction between sex and phenotype was detected.

Supplementary Figure S3

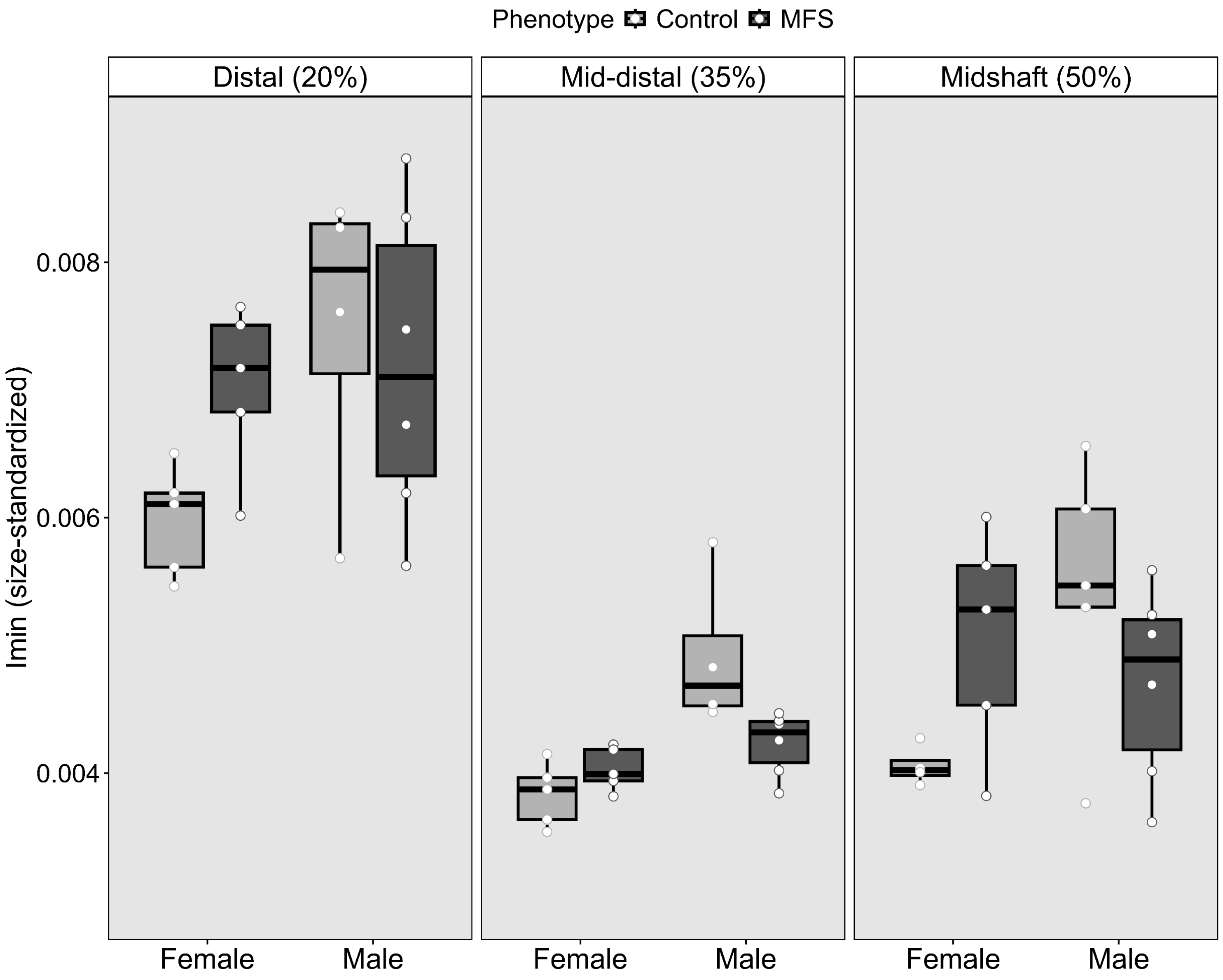

Figure S3. Size-standardized minimum second moment of area (IMIN) by sex and phenotype. MFS females have slightly higher minimum rigidity than controls, indicating greater resistance to bending stresses, whereas MFS males show reduced values. Black bars = medians.

Supplementary Fig S4. Examples of cortical bone in cross-section, one specimen per group

Female, Control (ID#1304)

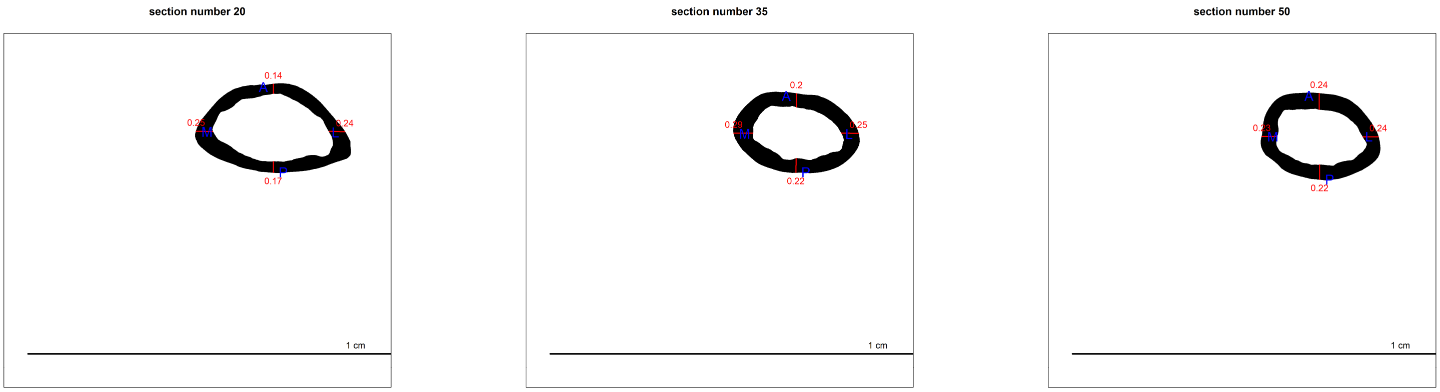

Female, MFS (ID#1300)

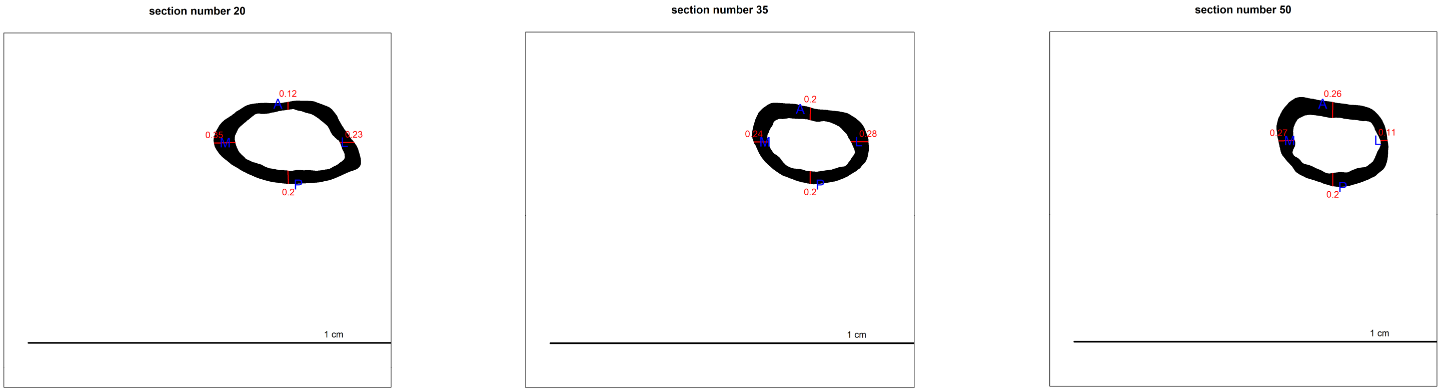

Male, Control (ID#1313)

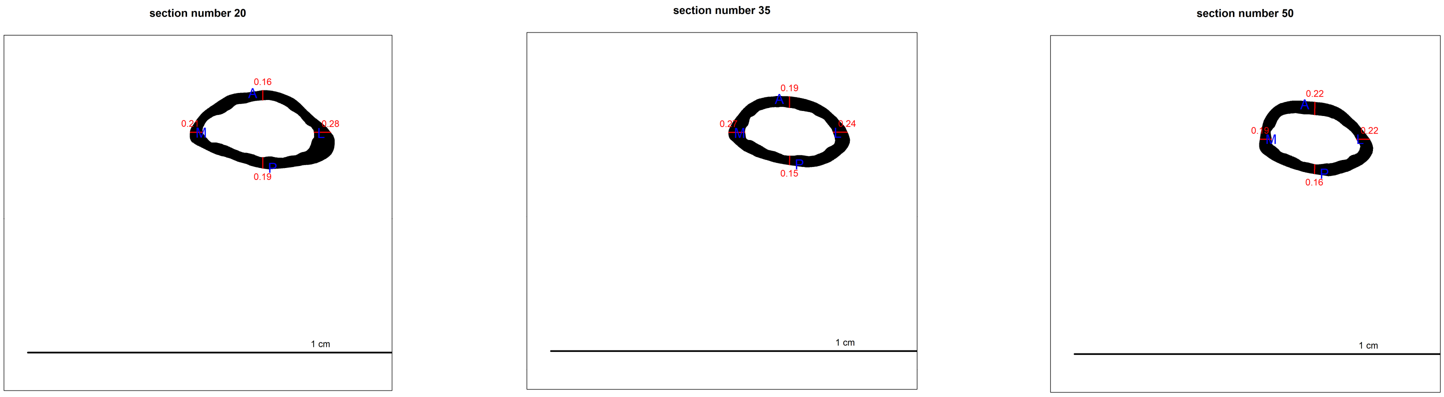

Male, MFS (ID#1310)

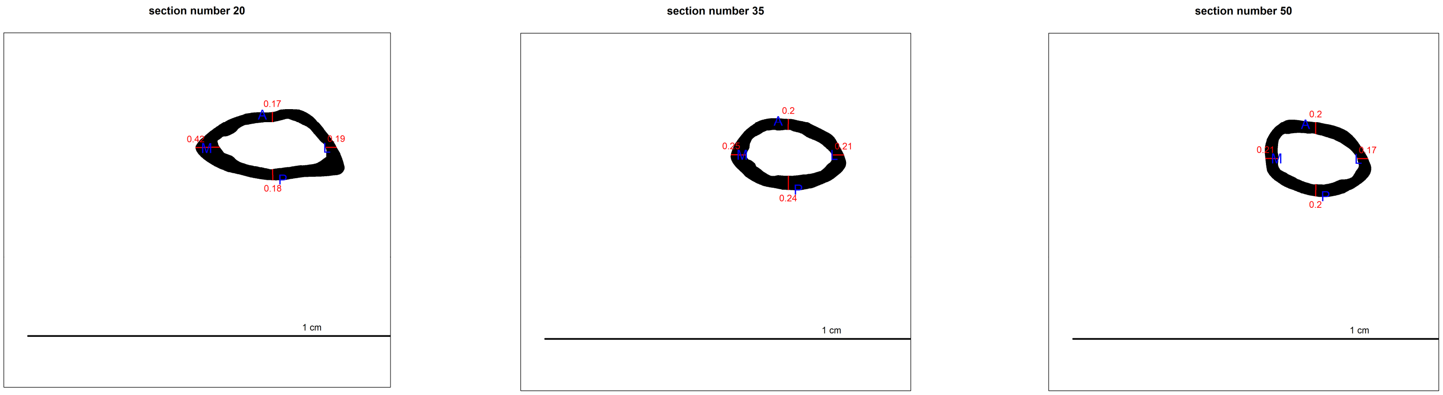

Supplementary Table S1

Table S1. Midshaft IMAX calculated from a single slice compared to the mean value of seven slices

Sex	Phenotype	Midshaft (single slice)	Midshaft (7 slices)
**Female**	Control	0.25±0.02	0.25±0.02
	MFS	0.28±0.02	0.29±0.03
**Male**	Control	0.44±0.12	0.44±0.10
	MFS	0.32±0.06	0.33±0.06

Mean ± SD. Results of a paired t-test were not significant (p-value>0.05)
